# A medium‐chain triglycerides‐enriched diet improves cognition and prevents metabolic and gut microbial alterations in Alzheimer’s disease models

**DOI:** 10.1002/alz70859_103337

**Published:** 2025-12-25

**Authors:** Paule E.H. M'Bra, Laura K Hamilton, Anne Aumont, Karine Prévost, Eric Massé, Karl JL Fernandes

**Affiliations:** ^1^ Université de Sherbrooke, Sherbrooke, QC Canada; ^2^ Université de Montréal, Montréal, QC Canada; ^3^ Université de Montréal/CRCHUM, Montréal, QC Canada; ^4^ Research Center on Aging (CdRV), Sherbrooke, QC Canada

## Abstract

**Background:**

Lifestyle‐based interventions can reduce 45% of dementia risk. Dietary supplementation with medium‐chain triglycerides (MCT) is a type of ketogenic diet that shows promise against Alzheimer’s disease (AD) in humans, presumably through hepatic conversion to circulating ketones. However, the physiological impacts and cellular mechanisms underlying MCT effects remain understudied, particularly in the context of AD.

**Objective:**

Here, we used two transgenic mouse model of AD to investigate the physiological and molecular mechanisms occurring in peripheral system upon an MCT‐enriched diet versus a classic ketogenic diet.

**Method:**

3xTg‐AD, 5xFAD mice and their respective control strain mice (WT) were administered at different age and duration, a standard carbohydrate‐rich diet (Control diet, 70% carbohydrate, 20% fat, 10% protein), a similar Control diet that was supplemented with ketogenic medium‐chain triglycerides (MCT, a ketogenic substrate), or an extreme carbohydrate‐free, high fat diet (CFHF). Mice were subjected to learning/memory tests, and longitudinal monitoring of body composition, glycemia, ketonemia and fecal microbiome composition.

**Results:**

Both ketogenic interventions improved cognition in AD mice after 1 month of treatment. Interestingly, unlike CFHF diet, MCT diet did not induce a sustained ketosis suggesting different mechanisms. Only the MCT diet improved peripheral glucose tolerance, insulin response and reduced adiposity, while CFHF dietary challenge exacerbated AD mice metabolic defects. AD mice exhibited several microbial alterations preceding cognitive symptoms, notably increased levels of *Bifidobacterium* and decreased levels of *Bacteroidetes*. Ketogenic interventions restored the fecal microbiome composition by 50% inducing a strong depletion of *Bifidobacterium*.

**Conclusion:**

Collectively, these findings reveal metabolism‐improving benefits of MCT in the context of Alzheimer’s disease that do not require elevated blood ketone levels and reveal potential therapeutic targets for treating AD, in the gut‐brain axis.

